# Commissural axon guidance in the developing spinal cord: from Cajal to the present day

**DOI:** 10.1186/s13064-019-0133-1

**Published:** 2019-09-12

**Authors:** J. D. Comer, S. Alvarez, S. J. Butler, J. A. Kaltschmidt

**Affiliations:** 1 Neuroscience Program, Weill Cornell Graduate School of Medical Sciences, New York, NY USA; 2 Developmental Biology Program, Sloan Kettering Institute, New York, NY USA; 3 Weill Cornell/Rockefeller/Sloan Kettering Tri-Institutional MD-PhD Program, New York, NY USA; 4 Department of Neurobiology, University of California, Los Angeles, Los Angeles, CA 90095 USA; 5 Molecular Biology Interdepartmental Graduate Program, University of California, Los Angeles, Los Angeles, CA 90095 USA; 6 Eli and Edythe Broad Center of Regenerative Medicine and Stem Cell Research, University of California, Los Angeles, Los Angeles, CA 90095 USA; 7 Department of Neurosurgery, Stanford University School of Medicine, Stanford, CA 94305 USA

**Keywords:** Floor plate, Ventral commissure, Decussation, Midline guidance cues, Commissural axons, Neurological diseases

## Abstract

During neuronal development, the formation of neural circuits requires developing axons to traverse a diverse cellular and molecular environment to establish synaptic contacts with the appropriate postsynaptic partners. Essential to this process is the ability of developing axons to navigate guidance molecules presented by specialized populations of cells. These cells partition the distance traveled by growing axons into shorter intervals by serving as intermediate targets, orchestrating the arrival and departure of axons by providing attractive and repulsive guidance cues. The floor plate in the central nervous system (CNS) is a critical intermediate target during neuronal development, required for the extension of commissural axons across the ventral midline. In this review, we begin by giving a historical overview of the ventral commissure and the evolutionary purpose of decussation. We then review the axon guidance studies that have revealed a diverse assortment of midline guidance cues, as well as genetic and molecular regulatory mechanisms required for coordinating the commissural axon response to these cues. Finally, we examine the contribution of dysfunctional axon guidance to neurological diseases.

## Introduction

The sensory and motor functions of the nervous system are central to the ability of an organism to sense and respond to the environment. These systems are inherently complex both due to the multiplicity of environmental stimuli and the extent to which an organism can sense and respond to them. The complexity of the nervous system is evident given neuronal population size and the degree of neuronal connectivity. The human nervous system is composed of over 10^11^ neurons, with each neuron capable of up to 10^4^ contacts, resulting in a monumental 1000 trillion synaptic connections. However, despite the seemingly overwhelming challenge of orchestrating the proper wiring of the nervous system during development, neuroanatomical studies have demonstrated a striking regularity in the arrangement of neuronal projections, a consequence of their tendency to compartmentalize in the formation of discrete neuronal fascicles and their precise guidance to their proper target regions. Extensive study of the manner in which developing axons traverse the developing central nervous system (CNS) has presented strong evidence for a molecular logic underlying the organization and guidance of neuronal axons during nervous system development.

**Fig. 1:**
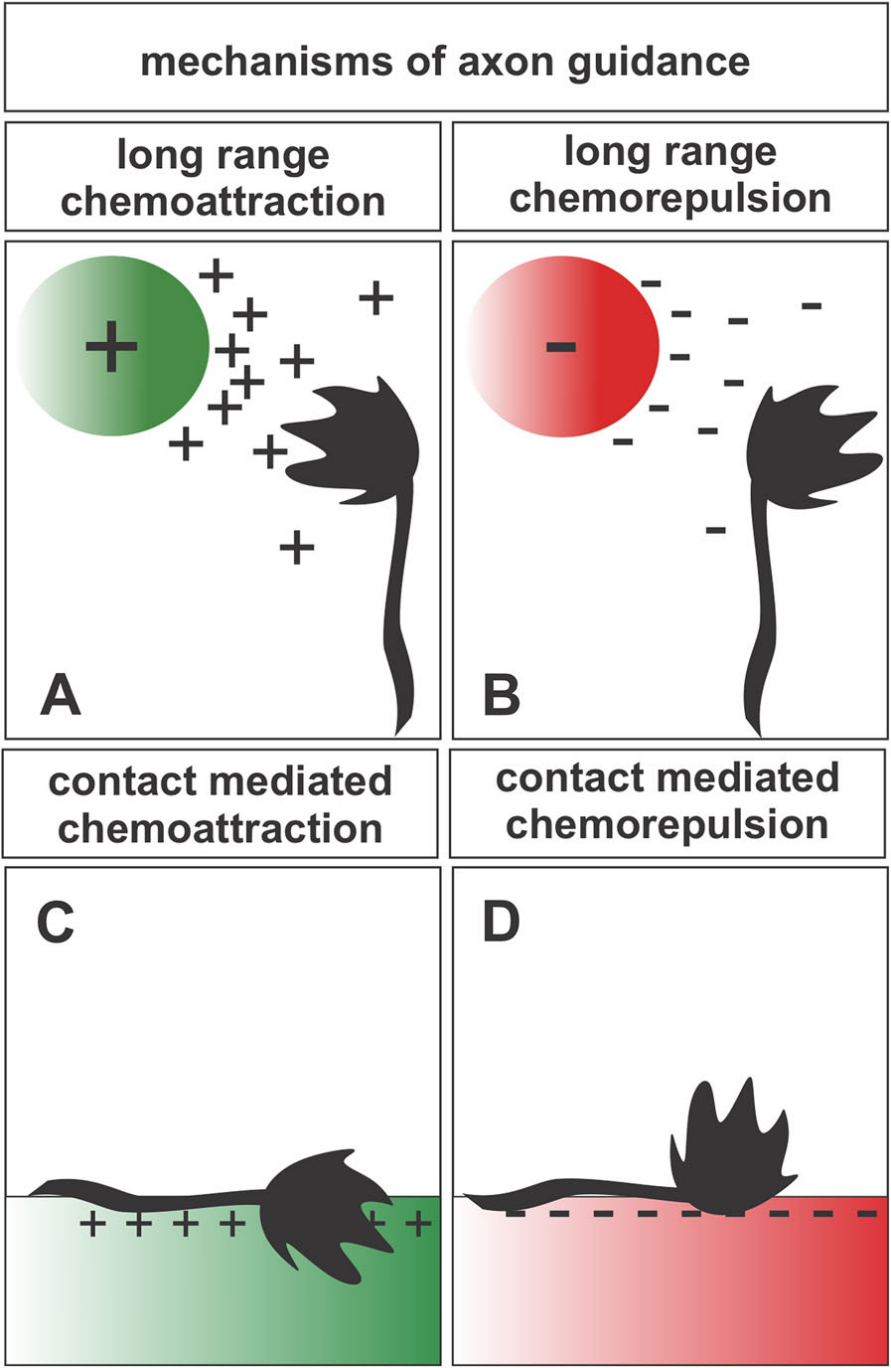
Mechanisms of axon guidance. Growth cones, the motile tip of a growing axon, integrate four major categories of guidance information [3]. **a**, **b** Long range chemoattractants (**a**) or chemorepellents (**b**) that act at a distance to orient the growth cone either towards or away from the signal. **c**, **d** Contact mediated chemoattractants (**c**) or chemorepellents (**d**) that orient axons through direct contact with the growth cone

Ramón y Cajal first proposed the directed development of axonal projections based on his studies in embryonic chick spinal cord and the observation of a specialized structure located at the tip of the developing axon, the growth cone [1]. In his neurotropic theory, Cajal considered the growth cone a dynamic, chemical sensing structure, responding to attractive substances provided by axonal targets, and ultimately guiding developing axons along a highly-stereotyped pathway “without deviation or error” [1, 2]. Remarkably in line with Cajal’s original observations, more recent studies have shown that developing axons navigate the primordial neuronal environment by detecting extrinsic molecular guidance cues that are presented to guidance cue receptors in the growth cone. By sampling guidance cues within the local environment, the growth cone steers axonal outgrowth in the appropriate direction, ensuring that the developing axon arrives within its intended target region. Although Cajal primarily considered the presentation of attractive substances to guide growth cone advancement [2], axon guidance studies have since expanded the diversity of molecular guidance cues to include both long-range and short-range contact-mediated chemoattractive and chemorepulsive cues (Fig. 1) [3]. Extensive studies of axon guidance mechanisms have identified four primary families of guidance cues – the netrins, slits, semaphorins, and ephrins – as well as guidance roles for other classes of molecules including morphogens, growth factors, glycoproteins, and cell adhesion molecules (CAMs) [3–6].

The axonal target is not the lone source for guidance substances as proposed by Cajal [2]. Rather guidance cues are additionally presented at intermediate points that lie along the axonal trajectory, effectively partitioning the pathway of a developing axon into a series of intermediate targets that orchestrate axonal arrival and departure [3]. A canonical example of a critical intermediate target is the floor plate (FP), which resides at the ventral midline and coordinates the midline crossing of commissural neurons at all levels of the CNS. The study of this midline crossing event has revealed fundamental molecular mechanisms of axon guidance in the developing CNS. These mechanisms include commissural axon guidance by attractive and repulsive axon guidance cues, as well as more recent evidence of the multifunctionality of guidance cue receptors [7, 8], commissural axon mutant phenotypes suggestive of undiscovered guidance cue receptors awaiting discovery [9–11] and a renewed interest in the contribution of long range versus short range signaling [12–19].

In this review, we will begin by considering the general question as to why commissural projections are such a predominant neuroanatomical feature. We will then discuss the studies of axon guidance mechanisms that are relevant for commissural axon midline crossing, focusing particularly on the netrins, slits, and their growth cone receptors. While these mechanisms are widely applicable to commissural neurons in the developing CNS, we will also consider other commissural neuron populations that appear to use alternate mechanisms to cross the CNS midline, including commissural neuron populations in the forebrain and those that cross at the dorsal midline of the spinal cord. To provide a more complete picture of the role of the CNS midline in neuronal development, we will also briefly discuss ipsilaterally-projecting populations. Finally, we will explore how genetic dysfunction of genes implicated in axon guidance manifest in neurological diseases.

### Contralateral projections and theories of decussation

Commissures are a common organizing principle found throughout the CNS, such that midline-crossing axons are a predominant neuroanatomical feature. Is there a functional or evolutionary advantage to this bilateral connectivity? At first glance, contralateral projections appear to be an obvious consequence of organism bilaterality and the need to coordinate sensory and motor function across the body. In the spinal cord, a prototypical example of bilateral motor control is the locomotor central pattern generator (CPG) that relies on commissural projections that cross at the ventral midline and contribute to the ventral commissure [20]. The CPG is comprised of commissural neuron populations from the V0 and V3 neuronal lineages, and loss of this bilateral connectivity disrupts the left-right rhythmicity required for locomotion [21, 22].

However, the purpose of midline crossing in other commissural neuron populations is less clear. The corticospinal tract (CST) is composed of cortical layer V pyramidal neurons. It crosses the CNS midline in the caudal hindbrain at the pyramidal decussation (a crossed tract of nerves) while *en route* to the spinal cord, where it ultimately activates spinal circuits for the initiation of voluntary movements. Proprioceptive and tactile information also projects to the contralateral CNS via secondary neurons in the caudal hindbrain that cross as internal arcuate fibers to form the medial lemniscus. This organization scheme results in the contralateral cortical processing of sensation and motor control, but it remains unclear why this neuroanatomical arrangement is present in the CNS and whether this arrangement was selected for according to functional advantage or evolutionary favorability.

#### Cajal and the first observed ‘decussation’

Most theoretical discussions of midline crossing in the CNS begin with the observation that the first ‘decussation’ occurs outside of the CNS at the pupillary eye, where the visual representation of the external environment becomes optically transformed as in a pin-hole camera, resulting in an inverted image at the retina [23]. Consequently, the internal representation of the external environment becomes flipped: left becomes right, and top becomes down [23]. Cajal was one of the earliest investigators to hypothesize that retinal ganglion cell (RGC) decussation at the optic chiasm compensates for this optical transformation at the eye. Schematically illustrating this phenomenon in lateral-eyed organisms [24], Cajal reasoned that the optic chiasm serves to align the two discontinuous retinal projections to produce an aligned, continuous internal visual representation. Further, he reasoned that in frontal-eyed organisms, such as humans, the partial overlap in retinal projections of the two eyes required that only the nasal retina cross at the optic chiasm [24], resulting in an optic tract composed of both contralaterally- and ipsilaterally-projecting RGCs. Because the reconstructed image is still necessarily inverted due to the optics of the eye, Cajal proposed that the sensorimotor systems must also compensate by crossing the CNS midline to ensure that both motor commands and sensory information are routed properly to be consistent with both the internal and external representations of the visual world (de Lussanet and Osse, 2012; 24). Additionally, this organization would permit visual central synapses to be in close proximity to motor and sensory circuits corresponding to the appropriate side of the body, resulting in decreased central reaction times in response to changes in visual stimuli [23].

Although Cajal’s theory remains one of the most compelling functional explanations for decussations at the optic chiasm and elsewhere in the CNS, some findings have challenged this model. Cajal hypothesized that decussation at the optic chiasm is needed for a continuous internal visual representation of the external environment. However, patients with non-decussating retinal-fugal fiber syndrome, where the optic chiasm does not form and all retinal projections are ipsilateral [25], show surprisingly normal visual processing despite the loss of binocularity [26]. It remains unclear whether interhemispheric pathways provide continuity between the two visual fields, or, more critically, if a continuous visual representation of the external environment normally occurs at all (de Lussanet and Osse, 2012). Additional examples that deviate from Cajal’s theory include the blind mole rat, which lacks an external eye and has a poorly defined visual field. Nonetheless, contralateral retinal projections are retained [27, 28], despite there being no obvious need for them.

#### An embryological twist and CNS decussation

Additional theories of decussation have offered functional hypotheses, including the facilitation of escape behavior [29] and the organization of neuronal information [30], while, other theories have considered decussations as a byproduct of early embryological morphological changes, i.e. not imparting any functional or evolutionary advantage. For example, to explain the decussation at the optic chiasm, de Lussanet and Osse proposed that, following a 90° turn about the body axis to the left side, two developmental compensatory rotations occur to regain bilateral symmetry, leading to a twist in the nervous system at the boundary between the forebrain and the midbrain [31, 32]. In addition to twisting the nervous system at this juncture, the forebrain is also inverted relative to the more caudal body parts [31]. Following this morphological change, the optic tracts develop and are guided toward the optic tectum. Assuming that the optic tracts preferentially target the optic tectum proximal to the retina prior to the morphological changes, de Lussanet and Osse argue that the optic tracts must cross the midline to contact the contralateral tectum to maintain this preferred connectivity, thus forming the decussation at the optic chiasm [31]. An additional theory of the formation of the decussation at the optic chiasm suggests a similar early embryological morphological change that results in a 180° somatic twist and the dorsal migration of the neuraxis [33].

In the formation of the decussation at the optic chiasm, both theories rely on the ability of developing RGC axons to sense ‘sidedness,’ requiring that RGC axons are capable of distinguishing between the ipsilateral and contralateral optic tectums. Several lines of evidence, however, demonstrate that commissural neurons do not necessarily exhibit an inherent preference for a contralateral target versus its mirrored, ipsilateral target. For example, despite disruptions in midline crossing of the RP3 and V motor neurons in *Drosophila*, these motor neurons are still able to properly respond to local, ipsilateral guidance cues to project to their mirrored target muscles on the ipsilateral side [34]. Further, in mutant mice, in which midline crossing from ventral cochlear neurons and inferior olivary neurons is lost, these neurons remain capable of projecting to their corresponding ipsilateral targets in the medial nucleus of the trapezoid body and cerebellum, respectively [35–37]. Together, these studies suggest that commissural axon guidance is not dependent on the position of the target neuron, but rather that specific interactions between commissural axons and the CNS midline are required for midline crossing to occur.

#### Decussated pathways and robust network design

Studies of commissure formation have revealed remarkably conserved molecular mechanisms of axon guidance that coordinate midline crossing [38]. Thus, rather than being a byproduct of an embryological formation event, could midline crossing instead represent a foundational feature of neuroanatomy? Further, could the topology of midline crossing impart an advantage during formation of the CNS? To address this question of topology in the wiring of the CNS during development, Shinbrot and Young used a computational approach to evaluate the network structure of the nervous system by considering multiple three-dimensional network topologies, including networks based on ipsilateral and decussated pathways [39]. With increasing network complexity, they found that a decussated arrangement minimized both miswiring events as well as the informational, or genomic, content required for network development [39]. These advantages may explain why decussated pathways are such a prominent neuroanatomical feature in both vertebrate and invertebrate nervous systems, and may underlie the evolutionary conservation of midline crossing due to the reduced susceptibility to miswiring events that it imparts during development. Even in smaller networks, decussation reduces miswiring events relative to other topologies, which may explain why an elementary decussated tract is present in *Caenorhabditis (C.) elegans* [39]. Interestingly, Shinbrot and Young observe that crossed pathways in *C. elegans* present an example in which decussation preceded the formation of complex visual organs, contrasting with the theory of decussation proposed by Cajal that stems from the organismal perception of the visual world.

### Commissural axons are directed towards the ventral midline of the CNS

The formation of functional neural circuits during development requires that axons can properly sense and respond to axon guidance cues within the extracellular environment to navigate towards their appropriate postsynaptic partners. Initial evidence that axonal pathways are partitioned into a series of steps came from axon guidance studies in grasshopper embryos and observations of the trajectories of pioneering neurons. Extensions from these earliest differentiating neurons traverse the developing CNS and provide a scaffold for the construction of subsequent neuronal circuits [40]. Observations of pioneering neurons in the migratory locust, *Locusta migratoria*, showed that early differentiating neurons in the peripheral sense organs and thoracic limb buds project highly stereotyped axonal trajectories toward their central targets [41, 42], suggesting that these pioneering axons used extrinsic cues derived from “guidepost” cells, found at consistent intervals along their path [41]. Ablating these cells resulted in pathfinding errors [43], supporting the hypothesis that guideposts cells were intermediate targets for pioneer axons. While the axons of later differentiating neurons were observed to merge with the axons of pioneering neurons [41, 44], they can also reach their appropriate target regions independently of this pioneering axonal scaffold [45, 46]. Thus, the ability to respond to axon guidance cues appears to be shared among developing neuronal populations and likely remains relevant throughout development and postnatal maintenance.

**Fig. 2:**
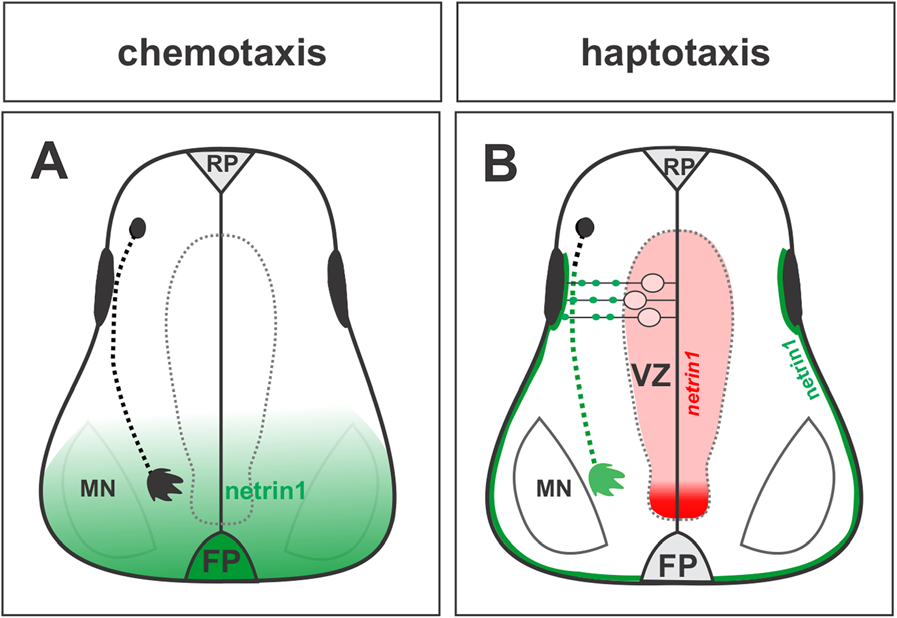
Comparison of netrin1 axon guidance models. **a** In the chemotaxis model, netrin1 acts as a long-range guidance signal. Commissural axons grow towards a diffusible source of netrin1 protein (green) emanating from the floor plate (FP). **b** In the haptotaxis model, netrin1 acts as a short-range guidance cue. Neural progenitor cells (NPCs) in the ventricular zone (VZ) express *netrin1* transcript (red). Netrin1 protein (green) is trafficked to the pial surface along the radial processes of the NPCs to form a growth substrate. Commissural axons extend along this netrin1 substrate, themselves accumulating netrin1 as they grow around the VZ towards the FP. Figure adapted from [13]

Similar to guidepost cells in the insect embryo, the columnar ependymal cells that comprise the FP at the ventral midline in the vertebrate embryo have been proposed to act as an intermediate target, guiding spinal commissural populations along their trajectory to the contralateral side of the CNS [47]. Studies over the past twenty years examining how commissural axons cross the ventral midline have revealed an intricate interplay between secreted (chemotropic) guidance cue expression by the FP and the regulation of commissural axon responsiveness to these cues [5]. Recent studies have additionally suggested contact mediated (haptotactic) mechanisms that coordinate the arrival of commissural axons to the ventral midline (Fig. 2) [12–15].

#### Evidence for the chemotaxis model of netrin1 function

The first direct evidence of chemotropism in the CNS came from in vitro experiments using embryonic rat spinal cord, which suggested the presence of a FP-derived axon guidance cue capable of directing commissural axon outgrowth toward the ventral midline (Fig. 2a) [48, 49]. When tissue explants taken from the dorsal-most spinal cord were co-cultured adjacent to FP explants, commissural axons, identified according to their expression of transient axonal glycoprotein (Tag)1 [50] grew in a directed manner towards the FP [48, 49]. Commissural axon outgrowth could also be simulated by culturing dorsal spinal cord explants in FP-conditioned medium [49], suggesting that the FP secretes a chemoattractant that directs commissural axons towards the ventral midline. In vivo evidence of this FP-derived chemoattractant was observed in the embryonic chicken spinal cord, when commissural axons reoriented their projections toward grafts of ectopic FP [51]. Further in vivo studies in zebrafish [52] and mouse [53–55] embryos demonstrated commissural axon pathfinding defects as they grow towards the ventral midline in genetic mutations preventing FP development.

The molecular identity of the FP-derived chemoattractant was discovered by systematically screening tissues to find a factor that could mimic the outgrowth promoting activity of FP conditioned medium [56]. Protein purification of chicken embryonic brain homogenate revealed two proteins, netrin1 and netrin2, that could promote the outgrowth of spinal commissural axons [56]. In situ hybridization experiments in chicken embryos demonstrated that the *netrin1* transcript was present in the FP, while *netrin2* mRNA was present in the ventral half of the neural tube [57]. The netrins exhibited homology with the *unc6* gene product [58, 59], previously shown to guide circumferential pioneering axons in *C.elegans*. Two homologs, netrinA and netrinB, were later also identified to play axon guidance roles in *Drosophila* [60, 61].

In vivo evidence for netrin1 acting as a long-range chemoattractant came from analyses of *netrin1* mouse mutants, which included both a hypomorphic allele, identified using a β-galactosidase-encoding gene trap approach [62] and a null mutation [63]. In embryonic day (E) 11.5 *netrin1* mutant spinal cords, Tag1^+^ commissural axons stall above the motor column, with the majority failing to cross the FP [13, 17, 63–65]. These results suggested that netrin1 was providing a long distance chemotropic signal to attract commissural axons toward the ventral midline [57]. Subsequent studies have shown that roundabout (Robo)3^+^ commissural axons and neurofilament (NF)^+^ axons, defasciculate in the absence of *netrin1*, growing both dorsally and into the ventricular zone (VZ) [13, 17]. Axon guidance defects were also present in the major commissures of the *netrin1* mutant forebrain, including the corpus callosum, and hippocampal and anterior commissures [64], however, notably the habenular and posterior commissures in *netrin1* mutants remain intact [64]. More recent, conditional genetic approaches have been used to probe netrin1 function in specific compartments of the spinal cord (see also section below). Removing netrin1 from the FP (netrin1ΔFP), results in the defasciculation and misrouting of Robo3^+^ commissural axons in the ventral spinal cord, again consistent with a long-range activity for FP-derived netrin1 [17, 19].

Netrin1 has also been suggested to elicit repulsion for various neuronal populations during development, including trochlear motor axons [66] and sensory axons as they enter the dorsolateral region of the spinal cord [67, 68]. Thus, the absence of *netrin1* transcript in the dorsal-most spinal cord may in part permit sensory commissural axons to enter and cross to the contralateral spinal cord [9, 69–72].

#### Evidence for the haptotaxic model of netrin1 function

Netrin1 has also been suggested to play a haptotactic role, defined as the directed growth of cells along an adhesive surface [73], or in response to substrate-bound cues [57], at both spinal and hindbrain levels, acting locally to guide commissural axons [12–15, 57]. A key to understanding netrin1 function lies in the differential distribution of its transcript versus protein (Fig. 2b). In situ hybridization studies in chicken first demonstrated that *netrin1* transcript is localized to the FP from early stages of development [57]. However, another member of the netrin family, *netrin2* is expressed by neural progenitor cells (NPCs) in the VZ of the spinal cord [57]. In mice, *netrin2* is not expressed in the spinal cord; rather the expression pattern of *netrin1* appears to be a composite of chicken *netrin1* and *netrin2*. Thus, by the stage at which the commissural axons begin their trajectory to the midline, *netrin1* transcript is present at high levels in both the FP cells and NPCs in the ventral two thirds of the spinal VZ [64], a region avoided by commissural axons [13]. In contrast, the distribution of netrin1 protein is distinct from that of its transcript. At the stage when commissural axons first start their extension, netrin1 protein is present at highest levels on the pial surface of the spinal cord [74]. At later stages when the first commissural axons have crossed the FP, high levels of netrin1 protein are additionally observed in the FP and on the commissural axons themselves [13, 74, 75]. Recent studies clarified the relationship between the *netrin1* transcript and netrin1 protein in mouse. The distribution of netrin1 on the pial surface has been proposed to stem from the ability of the *netrin1*^*+*^ NPCs to transport netrin1 protein along their radial process to their basal endfeet, where then it is deposited onto the pial surface (Fig. 2b). This phenomenon has been observed in both the spinal cord [13] and hindbrain [14].

Due to its complex expression in the spinal cord, the spatial requirement for netrin1 in commissural axon guidance has been assessed using conditional genetic approaches in mouse embryos [13–15, 17]. In the spinal cord [13], *netrin1* expression was specifically removed from either the NPCs in the dorsal VZ (netrin1ΔdVZ), or from FP cells (netrin1ΔFP). In the absence of NPC-derived netrin1, commissural axons become locally defasciculated in the dorsal spinal cord and project dorsally towards the roof plate (RP), and medially into the VZ. The number of Tag1^+^ axons reaching the FP was profoundly reduced [13]. Similar results were independently observed in the developing hindbrain [14, 15]. These findings demonstrated a novel role for NPCs in the VZ as a key source of netrin1 supplying guidance activities for commissural axons. The dorsal pial-netrin1 substrate appears to act by haptotaxis to promote commissural axon extension and direct fasciculated growth around the VZ.

The more recent conditional genetic studies suggested FP-derived netrin1 was dispensable for axon guidance [13, 14], because Tag1^+^ axons project largely normally towards the ventral midline in the netrin1ΔFP mice. However, further analysis of the netrin1ΔFP mice revealed defasciculation of Robo3^+^ axons (discussed in the previous section), and more local perturbations as commissural axons reach and cross the FP [17, 19]. Recent studies also examined the effect of removing all NPC-derived netrin1 (netrin1ΔVZ) [19]. This manipulation did not result in phenotypes with the same severity as those observed in the *netrin1* mutant, arguing that FP-derived netrin is sufficient to guide commissural axons ventrally. While the interpretation of this study is complicated by the presence of dorsal NPC-derived netrin1 at early stages in the netrin1ΔVZ line, it seems likely that both NPC- and FP-derived netrin1 have key axon guidance activities for commissural axons. Ongoing research will resolve when and where netrin1 acts as a short-range vs long-range cue.

Studies in flies and vertebrates have also suggested that netrin1 has an additional guidance activity establishing boundaries [12, 76, 77]. In the vertebrate spinal cord, netrin1 appears to encourage axon growth specifically around a *netrin1*^+^ domain [12]. This boundary activity was called a “hederal” boundary, from the analogy of a wall supporting the growth of ivy (genus: *hedera*) that is not itself penetrated by the ivy. Commissural axons always respect the edge of the NPC-*netrin1*^+^ domain, to grow around the VZ, and then adjacent to *netrin1*^+^ cells in the FP. When a small region of *netrin1* expression was extinguished in the intermediate spinal cord, axons deviated from their normal trajectories to follow the new boundaries in *netrin1* expression [13]. At later stages in spinal development, new domains of *netrin1* expression emerge adjacent to the dorsal root entry zone, which also serve as boundaries for spinal axon growth. Thus, netrin1 may supply both an adhesive substrate along which axons can grow in a fasciculated manner, while also providing a border to delineate axon tract formation. The mechanism that mediates the hederal boundary is not known, although it may require the deposition of netrin1 on commissural axons, since only netrin1^−^ axons are observed to stray into the VZ [12].

#### Different netrin receptors mediate the responsivity of commissural axons

Vertebrate netrin1 receptors were first identified based on homology with their *C. elegans* counterparts. Unc40 was proposed to be the receptor mediating ventral migration toward sources of Unc6 [58, 78]. Cloning the vertebrate homologs of Unc40 identified deleted in colorectal cancer (Dcc), previously known for its role in human colorectal neoplasia [79, 80], and neogenin, also shown to play a role in axon guidance [81]. By mouse stage E11.5, *Dcc* is broadly expressed by spinal neurons, while Dcc protein decorates commissural axons as they grow around the VZ [13] and towards the FP [82]. Dcc mediates the major guidance activities of netrin1 for spinal commissural axons [81]. Netrin1-dependent commissural axon outgrowth can be inhibited in a dose-dependent manner in vitro by the addition of an antibody against Dcc [82]. Most compellingly, *Dcc* null mutant embryos exhibit all of the axonal outgrowth defects observed in *netrin1* mutants, including the complete defasciculation of NF^+^ and Robo3^+^ axons and their subsequent growth into the VZ [13], the stalling of Tag1^+^ spinal commissural axons and severe reduction or absence of commissures in the forebrain [83]. Intriguingly, a key role of Dcc may be to facilitate the transfer of netrin1 onto axons [13, 76]. Netrin1 is still found on the pial surface in *Dcc* mutants, but it is not present on axons [13].

The role of neogenin in commissural axon guidance has remained unresolved. *Neogenin* transcript was initially thought to be absent from commissural neurons [82]. However, neogenin protein has been subsequently shown to be present on commissural axons [84, 85] and has been proposed to act with Dcc to guide commissural axons towards the ventral midline in a netrin1-dependent manner [81]. Neogenin also appears to functionally substitute for Dcc in chicken commissural axon guidance [85]. However, it remains unclear to what extent neogenin is required for midline attraction of commissural axons in other regions of the CNS. The seemingly complementary expression patterns of Dcc and neogenin [82] suggest that these receptor proteins may be differentially required to mediate netrin1-dependent responses in distinct populations of commissural neurons.

An additional family of netrin1 receptors was also identified by homology with *C. elegans*, where the Unc5 protein is thought to mediate the repulsive activities of Unc6 [58, 86]. There are multiple homologs of Unc5 in vertebrates, including Unc5a, Unc5b, Unc5c and Unc5d, which can bind netrin1 [87, 88]. Unc5c mediates netrin1-induced repulsion in sensory neurons [67, 68]. The response of commissural axons may be modified by the complement of guidance cue receptor complexes in the growth cone. Unc5a and Unc5b can complex with Dcc [66, 89, 90], which can convert netrin1-mediated commissural axon attraction to repulsion in vitro.

#### Netrin1-independent guidance mechanisms are also critical for spinal commissural axon guidance

Commissural axons are never observed to cross the RP at the dorsal midline at spinal cord levels, which suggests additional mechanisms exist to orient spinal commissural axons. Commissural axons are directed initially ventrally in response to a RP-derived chemorepellent, mediated by the bone morphogenetic protein (Bmp) family [91]. In vitro tissue culture assays demonstrated that Tag1^+^commissural axons will reorient away from either RP explants or COS cell aggregates expressing *Bmp7*, which is present in the RP [91]. This reorientation activity is lost from RP explants taken from *Bmp7* mutant embryos and some axons are observed to cross the RP in *Bmp7* mutant embryos in vivo [92]. However, subsequent studies using mutations in the Bmp signaling pathway revealed that the key in vivo role of the Bmps is to control the rate at which commissural axons grow towards the FP [93, 94].

An opposing reorientation activity is provided by the morphogen sonic hedgehog (Shh), present in the FP [55]. In the absence of Shh signaling in vivo, either through the conditional deletion of smoothened [55] or through loss of Boc, a non-canonical Shh receptor [17, 95], commissural axons are defasciculated and invade the motor column, consistent with a long range attractive activity. However, commissural axons can navigate towards and across the ventral midline in the absence of a FP [13, 55, 96], suggesting Shh is not absolutely required for the ventral extension of commissural axons. In in vitro experiments, COS cells expressing Shh phenocopy the activity of FP explants, reorienting Tag1^+^ commissural axons towards them [55]. This reorientation activity is thought to be redundant with netrin1, because it is still observed in FP explants taken from *netrin1* mutants [64], but not when Shh signaling is blocked [55]. Recent studies have also shown that the loss of Boc, the receptor that mediates Shh guidance activities [95] exacerbates the loss of FP-derived netrin1 from the FP [17], further indicating a key combinatorial and redundant role.

Finally, there is evidence that alternate mechanisms exist to mediate commissural axon midline attraction. Dcc and neogenin can also act in netrin1-independent manners, binding other families of ligands. Neogenin binds the family of repulsive guidance molecules (RGMs) [97]. A Dcc interaction screen identified cerebellin4, a member of the C1q tumor necrosis family, as having a role guiding axons at the brachial plexus [98]. At later embryonic stages, a population of L1CAM^+^ axons extends towards the midline in the dorsal spinal cord independently of netrin1 signaling [9].

### Navigating the CNS midline repellent guidance cues

#### Ipsilaterally projecting axons avoid the midline

In vivo studies have demonstrated that some axons approach the ventral midline only to turn abruptly away to follow an ipsilateral trajectory [99], suggesting that the FP is also a source of repulsive axon guidance cues. In the mouse nervous system, many axonal projections remain strictly ipsilateral, including projections from spinal neurons from the V1 and V2 lineages [100–102] and the dIL^B^ lineage that contributes to the dorsal funiculus and dorsolateral fasciculus [103–105], as well as projections from RGCs that do not cross the optic chiasm, but rather contribute to the ipsilateral optic tract [106, 107].

The mechanisms used to develop and maintain ipsilateral projections include the Robo/slit, Npn/Sema, and Eph/ephrin families of repellent guidance molecules [103, 105, 108]. These repulsive signaling mechanisms appear to be controlled at the transcriptional level. Zic2 expression is shared by ipsilaterally-projecting retinal and dorsal horn neuronal populations [103, 105, 109, 110] and represses transcriptional programs required for midline crossing in the ventral spinal cord [103, 111]. Transcriptional repression of ipsilateral developmental programs has also been shown in the retina where the LIM-homeodomain transcription factor Isl2 represses the expression of Zic2 [112].

#### Contralaterally-projecting axons can change responsiveness to the ventral midline after crossing

**Fig. 3:**
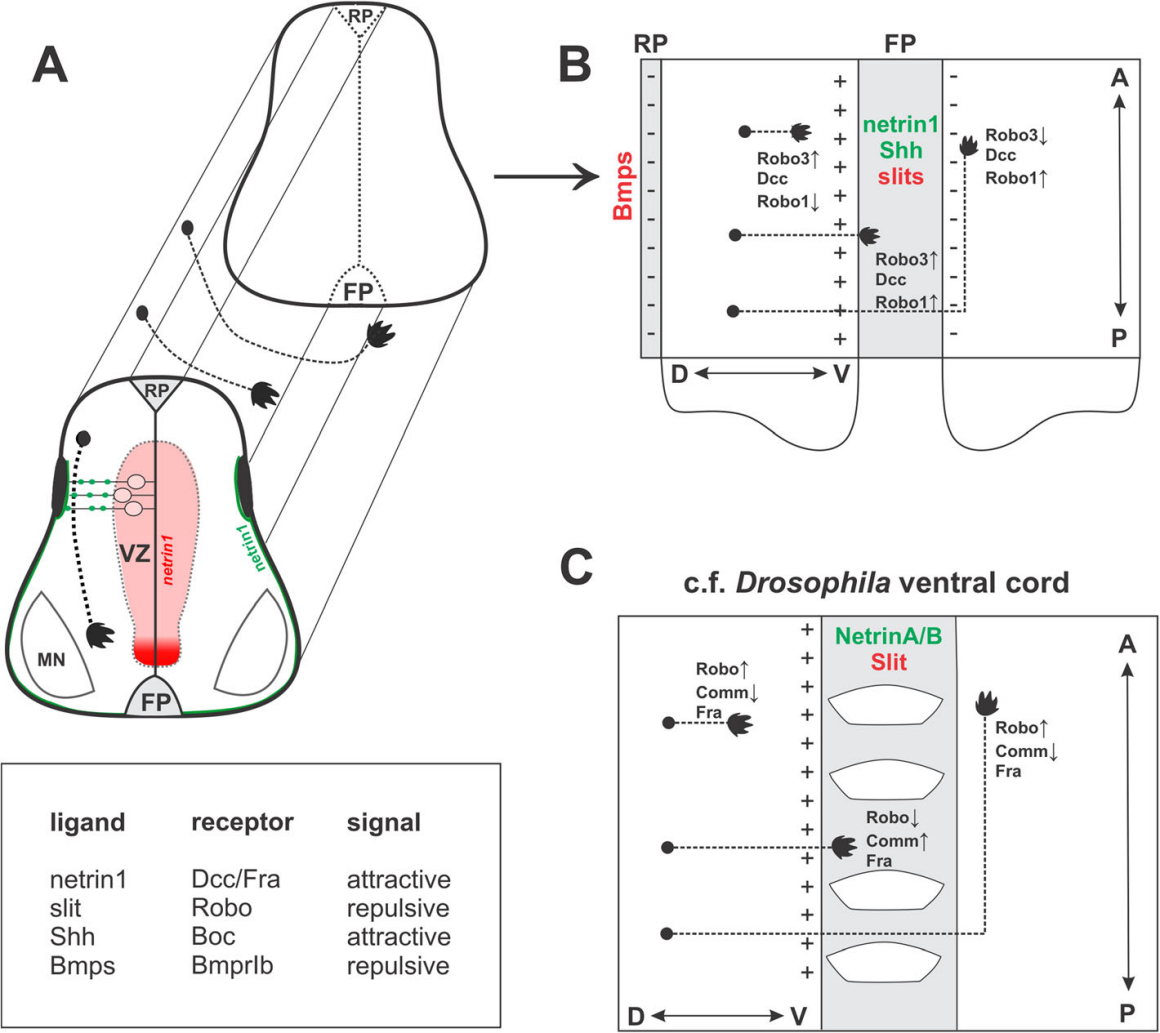
Summary of ventral midline crossing. **a** In the vertebrate spinal cord, dorsal commissural neurons extend their axons ventrally. They are guided first by the roof plate (RP)-chemorepellent, mediated by the Bmps acting through BmprIb, that directs them away from the dorsal midline. Commissural axons are then directed towards the floor plate at the ventral midline, considered a classic example of an axon guidance guidepost, by action of two attractants, netrin1 and Shh, in a Dcc- and Smo/Boc-dependent mechanism, respectively. **b** Midline crossing is mediated by the slit/Robo pathway. The floor plate (FP) expresses the slit repellent, which is detected by the robo receptor family. Pre-crossing commissural axons are unresponsive to slit, as a consequence of the expression of Robo3, which interferes with Robo1 function. However, after crossing the midline, Robo3 expression is downregulated, such that Robo1^+^ commissural axons become sensitive to the presence of slit, guiding the axons away from the midline and preventing the axons from re-crossing the midline. **c** Guidance decisions are largely conserved in the *Drosophila* nerve cord. Attraction of commissural axons to Netrin1 is mediated by the Dcc homologue Frazzled (Fra). Similarly, Slit proteins regulate behavior of pre and post-midline crossing Robo^+^ axons through repulsive signaling. However, in *Drosophila*, Robo levels are regulated by Comm, which endocytoses Robo in axons, making them unable to detect slit repulsion, and thereby permits axons to cross the midline

Unlike ipsilaterally-projecting neurons, commissural neurons grow towards, cross, and exit the CNS midline (summarized in Fig. 3). One explanation for this behavior is that commissural axons modulate their responsiveness to attractive and repulsive guidance cues by altering the spatial distribution of axon guidance receptors [10, 113–116], allowing commissural axons to change their responsiveness to guidance cues over time. Tag1^+^ spinal commissural axons appear to lose responsiveness to netrin1 and Shh after crossing the midline [117, 118], thus preventing post-crossing commissural axons from being persistently attracted to the midline. Additionally, the responsiveness of commissural axons to midline-derived repulsive cues is also altered during midline crossing [119]. Members of the semaphorin [120] and slit [121] families can elicit commissural axon repulsion in vitro, and may play critical roles during commissural axon midline crossing in vivo. Here, we will focus on the slit/Robo family of ligand and receptors and how commissural axons may alter their responsiveness to slit repulsion during midline crossing using a mechanism dependent on the divergent Robo family member, Robo3/Rig1.

The *slit* gene was originally discovered in *Drosophila,* in a screen of embryonic lethal mutations with segment abnormalities in the larval cuticle [122]. *Slit* is expressed by glia at the CNS midline [123, 124]. In the absence of *slit*, midline glia are displaced from the nerve cord [125] and there is a profound disturbance in the segmentally-repeating array of commissural nerves, normally present in *Drosophila* wild type embryos. The commissures collapse, leaving only a single longitudinal axonal tract at the midline [123, 124]. A similar commissural axon guidance phenotype was reported in the *single-minded* (*sim*) mutant: the midline glia do not form, and commissural axons accumulate at the CNS midline [126, 127]. Together, these studies underscored the critical role midline glia may have organizing commissural axon tracts at the CNS midline and further suggested *slit* as a key midline repellent.

A genetic screen identified two further genes required for commissural axon organization: *commissureless* (*comm*) and *robo*. During development in *comm* mutant embryos, commissural axonal outgrowth is initially normally oriented toward the midline [128]. However, this midline-directed axonal outgrowth eventually stops, and commissural axons turn before crossing the midline to inappropriately join the longitudinal connectives on the ipsilateral side [128], thereby resulting in the loss of the commissures. In contrast, in the *robo* mutant, ipsilaterally-projecting neurons acquire the ability to cross the midline, while commissural neurons now recross multiple times, resulting in thick commissures and minimal longitudinal connectives [115, 128]. Importantly, in both mutations the midline glia develop normally [128], which suggested that the phenotype is due to a primary defect in axon pathfinding rather than secondary defect in midline glial development or differentiation. Further, despite inappropriate midline crossing, axons in both *comm* and *robo* mutant embryos are able to appropriately reach their mirror image equivalent synaptic targets [34], suggesting that they function specifically in growth cone guidance, rather than synaptogenesis.

Robo was proposed to participate in a repulsive signal that prevents axons from crossing the midline, based on the observation that ipsilaterally-projecting axons make inappropriate contralateral extensions in *robo* mutants [128]. Since the *comm; robo* double mutant phenotype is strikingly similar to that in the *robo* mutant alone, Comm was proposed to function upstream of Robo, regulating its function to orchestrate midline crossing [128]. However, direct evidence of how *comm* functioned in relation to *robo*, remained elusive. Clues came from the expression patterns of Comm and Robo, which are tightly coupled with both respect to each other and the position of growth cone relative to the midline [115]. In control embryos, Robo protein is present at high levels on longitudinally-projecting axons to prevent them from crossing the midline, while its absence from commissural axons ensures that they only cross the midline once [115]. In *comm* hypomorphic alleles, Robo is present at higher levels on commissural axons suggesting that *comm* suppressed Robo levels on commissural axons [115]. In contrast, the consequence of overexpressing *comm* resembles the *robo* mutant phenotype: there are reduced levels of Robo protein in the commissural axons, which abnormally cross and recross the midline [115]. Similarly, forced expression of *comm* in ipsilateral neurons enables them to cross the midline [129].

Subsequent in vivo studies have suggested that Comm acts as an intracellular sorting receptor for Robo, intercepting it before reaching the growth cone in vivo [130–132]. Our understanding of Comm function, however, may still be incomplete; further studies have suggested a mechanism of Robo silencing by Comm that is sorting-independent [133]. More recent studies have shown that slit-dependent endocytosis of Robo receptors is required for Robo receptor activation [134].

A further advance in our understanding of commissural axon midline crossing came from the discovery that Robo is an evolutionarily conserved axon guidance receptor [114, 135, 136] and that slit binds the Robo receptor to elicit axon repulsion [10, 135, 137–139]. Studies in *C. elegans* identified *sax3* and *slt1* as the respective homologs of *Drosophila robo* and *slit* [136, 140]. Initial reports suggested that mammals had two *robo* homologs, *robo1* and *robo2*, and three *slit* homologs, *slit1*, *slit2*, and *slit3* [114, 135, 141]. Subsequent studies sought to determine whether these homologs had conserved function, permitting axons to navigate the CNS midline.

The distribution of the *robo1/2* and *slit1/2/3* transcripts, and Robo1/2 protein in the rodent embryonic spinal cord shows remarkable similarity to their counterparts in the *Drosophila* nerve cord. In rodent embryos, *robo1* and *robo2* transcripts are expressed in overlapping patterns in many populations of neurons, while the three *slit* transcripts are all present in FP [10, 114, 116, 135]. Both Robo1 and Robo2 are present at higher levels on the post-crossing segments of commissural axons [10, 116, 139], correlating with their acquiring sensitivity to the slit repellents following midline crossing [119]. Together, these observations supported the model that the upregulation of Robo1/2 in post-crossing commissural axons permits them to recognize the slit repellent in the ventral midline, and thereby avoid it.

Mouse mutant studies also supported a role for slit/Robo signaling in midline crossing. Commissural axons stall or re-cross the ventral midline in *slit1; slit2; slit3* triple mutants [10]. *Robo1*, *robo2*, and *robo1;robo2* mutants demonstrate axon stalling and recrossing defects at the ventral midline, as well as defects in axon sorting in the ventral and lateral funiculi [10, 11]. Thus slit/Robo signaling appears to function similarly in both vertebrate and invertebrates: slit is required to expel Robo1/2^+^ commissural axons from the ventral midline and thereby prevent them from re-entering the midline. The axon guidance phenotype in *robo1/2* double mutants is less severe than that in s*lit1; slit2; slit3* triple mutants, suggesting that commissural neurons may possess an additional receptor for slit [10, 11]. Moreover, the *robo1/2* double mutant pathfinding defect in spinal commissural neurons that normally cross at the dorsal midline [9] may unmask the activity of an additional slit receptor responsible for this dorsal midline repulsion.

While the vertebrate slit and Robo family members demonstrate many functional similarities with their *Drosophila* homologs, no vertebrate homolog of Comm has been identified. However there are multiple candidates for functional homologues, these candidates include 1) the WAGR syndrome PRRG4, which can re-localize Robo away from the cell surface in vitro [142], 2) Rab guanine nucleotide dissociation inhibitor (GDI), which regulates the levels of Robo1 on commissural axons in the chicken spinal cord by controlling its insertion into the growth cone membrane [143] and 3) two Nedd4-interacting proteins, Ndfip1 and Ndfip2, that localize Robo1 to endosomes [144]. Alternative mechanisms have also been suggested for vertebrate commissural axons to regulate their responsiveness to the slit repellent. A critical clue to this regulation came from the identification of Robo3 (Rig1), a third member of the vertebrate Robo family. Robo3 was first identified as a factor that is upregulated in *Retinoblastoma* mutant embryos [145]. It was subsequently found to be defective in humans exhibiting uncrossed sensory and motor projections in the hindbrain [146]. *Robo3* mutant mice similarly display a lack of commissures in the hindbrain, and at the ventral midline throughout the developing spinal cord [116, 147]. The distribution of Robo3 inversely correlates with Robo1 and Robo2: it is only present on pre-crossing commissural axons [116]. This expression pattern raises the possibility that Robo3 interferes with Robo1/2 to prevent slit-mediated repulsion in commissural axons prior to midline crossing. Supporting this model, pre-crossing commissural axons fail to cross the ventral midline in *robo3* mutants, and follow an ipsilateral pathway [116], suggesting that they are prematurely responsive to slit repulsion. However, Robo1 and Robo2 protein expression is not upregulated in pre-crossing axons in *robo3* mutants [116], suggesting that Robo3 functions differently from *Drosophila* Comm. Partial rescue of the *robo3* phenotype is seen in *robo1; robo3* and *robo1; robo2; robo3* double and triple mutants [11, 113, 116], suggesting that Robo3 inhibits Robo1 and Robo2 receptor function on pre-crossing axons, but does so using a mechanism that does not alter the distribution of Robo1 and Robo2 protein. The molecular basis of this mechanism remains unresolved. One possible role for Robo3 on precrossing segments would be to bind and sequester slit protein to prevent repulsion [116], however recent studies have shown that Robo3 does not bind with slit with high affinity [8], making a signaling role more likely. A more recent cell culture study proposed that Robo3 does not bind slit protein but rather recruits Robo1 and Robo2 into an endocytic pathway [148], possibly reflecting a function similar to *Drosophila* Comm. While *robo3* mutants display a striking loss of commissures at the ventral midline throughout the developing spinal cord and hindbrain [116, 147], major commissures in the forebrain persist despite an ongoing requirement for Robo and slit [149]. Thus, other mechanisms may regulate commissural axon responsiveness to midline-derived repellents.

### Axon guidance defects and human disease

Multiple human neurological disorders result from developmental errors in axonal pathfinding [150–152]. Here, we will focus on the two neurological disorders – horizontal gaze palsy with progressive scoliosis (HGPPS), congenital mirror movements (CMM) – that involve the axon guidance mechanisms discussed in the preceding sections.

#### Horizontal gaze palsy with progressive scoliosis (HGPPS)

HGPPS is a rare autosomal recessive disorder stemming from mutations in the *ROBO3* gene, which results in the loss of midline crossing in the hindbrain [146]. Human *ROBO3* mutations result in the complete loss of ROBO3 function [152], resulting in HGPPS patients tending to present similarly. They show an absence of congenital horizontal eye movement and the development of severe scoliosis in early life [153]. The failure of commissural axons to cross the midline in the hindbrain results in both 1) the ascending sensory axons of the dorsal columns-medial lemniscus pathway and 2) descending motor axons that comprise the corticospinal tract (CST) projecting ipsilaterally [146]. Imaging studies revealed that midline crossing is disrupted for the superior cerebellar peduncles and pontine axons, that normally project contralaterally through the middle cerebellar peduncles [154]. The auditory pathways are also compromised [155, 156]. Axon projection analysis in *robo3* mutant mice has shown a reduction in cochlear nucleus projections that normally cross the midline [36], suggesting that defects in this pathway may contribute to the auditory deficits observed in human disease.

The deficits in horizontal eye movement in HGPPS patients suggest that contralateral extraocular motor pathways are also affected, including contralateral inputs onto the abducens nucleus from the paramedian pontine reticular formation and projections from the abducens nucleus that target the contralateral oculomotor nucleus via the medial longitudinal fasciculus [150]. A HGPPS mouse study in which *robo3* was conditionally knocked out in the hindbrain supports this analysis by reporting a reduction in contralateral projections at the level of the abducens nucleus and marginal connectivity between the abducens and contralateral oculomotor nucleus [36]. The severe scoliosis that develops during childhood, however, is less well understood and is thought to involve asynchronous muscle contractions, which underlie the breathing deficits in *robo3* mutant mice [152, 157], as well as defects in axial motor control [146].

Despite defects in the formation of hindbrain commissures, a common feature in HGPPS patients is the persistence of commissures at other levels of the CNS, suggesting that ROBO3-independent mechanisms play a role in the formation of these commissures. For example, the major forebrain commissures appear to be intact in HGPPS patients, including the corpus callosum [146]. There is normal decussation at the optic chiasm [158], and the spinothalamic tract crosses at the ventral midline in the spinal cord [29]. Studies in *robo3* mutant mice have similarly reported the persistence of forebrain commissures [149] as well as commissures continuing to be present in the dorsal spinal cord [9]. Functionally, HGPPS patients generally perform well on neuropsychological testing and do not exhibit mirror movements [155], suggesting that a non-decussating CST alone is insufficient to produce mirror movements. Instead, the development of these mirror movements may require the contralateral sprouting of CST axons in the spinal cord as in Klippel-Feil syndrome [29], raising the intriguing possibility that ROBO3 may be required for this contralateral sprouting.

#### Congenital mirror movements (CMM)

CMM are involuntary movements that simultaneously accompany voluntary movements on the contralateral side of the body. They often occur as part of a neurological syndrome, including the Klippel-Feil, Kallmann, and Joubert syndromes [152, 159]. This dysfunction is thought to involve the inappropriate bilateral activation of primary motor cortex stemming from defects in the formation of the corpus callosum [160] and the CST, involving incomplete decussation within the hindbrain [161], abnormal persistence of ipsilateral CST projections [162, 163], and inappropriate contralateral branching within the spinal cord [164].

Because CMM are a symptom in a number of neurological syndromes that are likely to have mutations at multiple genetic loci, it has remained unclear which genetic mutations specifically result in CMM. However, defects in netrin1/Dcc signaling have recently been implicated as causal factors for CMM. First, genome-wide linkage analyses identified mutations in the *DCC* gene in two unrelated families with CMM. These mutations are predicted to result in either a truncated form of the receptor that cannot bind netrin1 [165], or a form that prevents DCC dimerization [152], resulting in its degradation by nonsense-mediate mRNA decay [166]. These studies also proposed that *DCC* mutations produce mirror movements because of inappropriate ipsilateral CST projections from the hindbrain [165, 166]. During mouse CNS development, Dcc is present in CST axons [167] and mutations in *Dcc* disrupt the CST at the pyramidal decussation [168]. Further, in *Dcc*^*kanga/kanga*^ mutant mice, which are viable to postnatal ages [83, 168], the hindlimbs move synchronously in a hopping gait [168], recapitulating the mirror movements seen in patients with *DCC* mutations.

Until recently, mutations in the *NETRIN* (*NTN*)*1* gene had not been directly linked to an inherited neurological human disease. However, exome sequencing studies have now identified three variants of *NTN1* in members of two unrelated families and an unaffiliated individual with CMM. The three variants, which include two missense mutations (Cys601Ser and Cys601Arg) and one in-frame deletion (Ile1518del), all localize to the netrin (NTR) domain found at the C-terminus of the protein. Through molecular modeling software Cys601 is predicted to be important for the formation of disulfide bridges, while Ile518 is part of a beta strand [169]. While the NTR domain is not necessary for secretion or binding to Dcc [170], the NTR mutations are predicted to cause structural changes that would affect the folding and subsequent processing of NETRIN1 [169]. In these cases, CMM appears to be a direct result of *NTN1* disruption and not a secondary consequence of a neurological syndrome. The patients do not have other observable neurological defects or mutations in any of the genes previously associated with CMM [171, 172]. A tractography analysis of the CST in the *NTN1* patients demonstrated that they have an increased proportion of ipsilateral CST projections compared to control subjects [169], suggesting a role for netrin1 regulating axons crossing the CST midline. In vitro studies have suggested that the mutant *NTN1* allele affects the localization and processing of netrin1 for secretion from the cell. HEK239 and Hela cell cultures were transfected with either the control or mutated allele of *NTN1*, cells were cultured, the supernatant was collected and the cells were lysed to collect the intracellular fraction [169]. A higher proportion of netrin1 was found in the intracellular fraction in the mutant cultures compared to controls. Together, these studies suggest that the *NTN1* exon 7 mutation reduces the level of netrin1 in the extracellular matrix, thereby leading to reduced or aberrant crossing of axons in the CST, resulting in CCM.

## Conclusion

Axon guidance studies have suggested a model in which developing axons traverse a sequence of intermediate targets during development. Navigating these intermediate targets requires that developing axons respond to extracellular attractive and repulsive guidance cues, including members of the netrin and slit families, which are provided by specialized populations of cells that reside along the axonal trajectory. Commissural neuron midline crossing has provided a valuable model for the study of axon traversal at the CNS midline intermediate target and has revealed evolutionarily conserved molecular mechanisms that underlie axon guidance. Interestingly, theories of decussation have suggested that midline crossing may have been evolutionarily selected for based on its property to minimize wiring errors during development, suggesting that axon guidance studies at the CNS midline may reveal some of the fundamental aspects of CNS development and organization. Of particular interest is the fundamental property of how commissural axons regulate their responsiveness to axon guidance cues so that developing axons appropriately extend from one intermediate target to the next without stalling or recrossing previous targets. Studies of these commissural populations will advance both our basic knowledge of axon guidance in the developing CNS as well as our understanding of how axon guidance defects lead to disease.

## Data Availability

Data sharing not applicable to this article as no datasets were generated or analyzed during the current study.

## Abbreviations

Bmp: Bone morphogenetic protein
*C. elegans*: *Caenorhabditis elegans*
CAMs: Cell adhesion molecules
CMM: Congenital mirror movements
CNS: Central nervous system
*comm*: *Commissureless*
CPG: Central pattern generator
CST: Corticospinal tract
Dcc: Deleted in colorectal cancer
dVZ: Dorsal ventricular zone
E: Embryonic day
FP: Floor plate
Fra: Frazzled
HGPPS: Horizontal gaze palsy with progressive scoliosis
NF: Neurofilament
NPCs: Neural progenitor cells
*NTN*: *NETRIN*
RGC: Retinal ganglion cell
RGMs: Repulsive guidance molecules
Robo: Roundabout
RP: Roof plate
Shh: Sonic hedgehog
*sim*: *Single-minded*
Tag: Transient axonal glycoprotein
VZ: Ventricular zone
